# Evaluation of the Properties and Microstructure of Thick-Walled Welded Joint of Wear Resistant Materials

**DOI:** 10.3390/ma15197009

**Published:** 2022-10-09

**Authors:** Ryszard Krawczyk, Jacek Słania, Grzegorz Golański, Adam Zieliński

**Affiliations:** 1 Faculty of Mechanical Engineering and Computer Science, Czestochowa University of Technology, Armii Krajowej 21, 42-201 Częstochowa, Poland; 2 Department of Material Engineering, Czestochowa University of Technology, Armii Krajowej 19, 42-201 Częstochowa, Poland; 3 Łukasiewicz Research Network-Institute for Ferrous Metallurgy, K. Miarki 12-14, 44-100 Gliwice, Poland

**Keywords:** wear-resistant steel, welded joint, weldability, microstructure, mechanical properties

## Abstract

The research was conducted on a thick-walled welded joint between the HTK 900H wear-resistant steel plates and the A6 cast profile. The aim of the experiment was to produce a joint with the relevant performance requirements, i.e., a good abrasion resistance joint in the weld face area while ensuring its proper plasticity. The welded joint was made using the MAG PULSE and the high-performance MAG TANDEM methods under automated conditions using the linear welding energy ranging from 1.2 to 2.2 kJ/mm for the different joint regions. The scope of the research included both non-destructive and destructive testing. The non-destructive visual (VT), magnetic-particle (MT), and ultrasonic (UT) tests revealed a good quality of the welded joint with no significant welding imperfections. The microstructure of the welded joint in the weld zone was characterized by a dominant volume fraction of martensite/bainite. The measurement of hardness near the face of the weld confirmed obtaining similar values for this parameter. The HTK 900H steel was characterized by hardness at the level of 383 HV10, whereas the A6 cast-328 HV10, and the weld-276 HV10. At the same time, the analyzed joint showed high ductility in the range of 86 to 159 J. The tests carried out showed that the linear energy control allowed a welded joint with the required performance characteristics to be obtained.

## 1. Introduction

The construction of modern equipment for the mining and processing industries requires the use of materials with specific wear resistance properties; this includes, but is not limited to, the components of mechanically combined cutter loaders, excavators and large-size bulldozers, aggregate crushing mills and equipment of the loading and transport systems used, e.g., in the mining and cement industry. For these applications, wear-resistant materials such as rolled plates and cast profiles are most commonly used. In the case of complex structural components, it is often necessary to join them using welding methods [1,2,3]. Therefore, there are additional requirements concerning the consideration of a number of important issues related to the weldability assessment of the materials to be joined, especially for thick-walled components. These issues include the selection of the proper welding method, filler metals, welding procedure, as well as heat treatment and method of joint preparation for welding [4,5]. The appropriate selection of welding conditions should ensure that a joint with properties similar to those of the materials being joined are produced [6,7,8,9]. Compliance with these requirements should provide the appropriate operating conditions for all components in the structure, including the joints used in the mining or processing equipment under construction. The suitability of the materials along with the joints used in the structures is determined by a set of technological properties related to the weldability assessment combined with the operational factors, which in this case are of priority nature and are closely related to the economic requirements [10,11,12]. The HTK 900H steel has relatively good weldability, low susceptibility to cold cracking and required strength and plastic properties of the welded joint, while ensuring high abrasive wear resistance [3,7,8,10,11]. Hardox steels can be divided into the following classes according to the Brinell hardness value: 400, 450, 500, 550, 600. The increase in Hardox steel hardness, i.e., the increase in its code number, means the higher hardness, strength properties and abrasion resistance, and at the same time, the deterioration in the plastic properties, toughness and weldability [3,7,10,11,12,13]. The research into the weldability of Hardox steel carried out so far concerned mainly the issues relating to the determination of the effect of the welding process parameters on the microstructure and mechanical properties of the joints produced, the effect of the filler metal on the properties of the joint and the ability to produce dissimilar joints, i.a. [7,10,11,13,14,15,16,17,18]. Technically, it is extremely important to be able to make dissimilar joints that allow these steels to be used in a number of structures [15,16,17,18]. The welded joints of this type have to meet the required abrasion resistance criterion, and at the same time, they must have the relevant plasticity and toughness within the heat-affected zone (HAZ). However, there is not enough data in the literature which presents the possibility of producing the correct dissimilar welded joint with dedicated properties without interrupting the welding process under automated conditions. In addition, thick-walled (t ~ 40 mm) dissimilar welded joints of Hardox steel have not been widely studied. The results of my own research presented in the article are to enhance the knowledge of the weldability of wear-resistant materials by evaluating the properties of a thick-walled welded joint of HTK 900H steel with an A6 cast profile.

## 2. Materials and Methodology

The material used in the research was a test welded joint, including wear-resistant materials. The joint was the connection of thick-walled elements (t = 40 mm) of wear-resistant HTK 900H plate with an A6 cast profile [19,20]. The characteristics of the welded base materials based on the manufacturer’s certificate are given in Table 1, while the characteristics based on the own chemical composition analysis with a SpectroLab spark spectrometer are provided in Table 2. The characteristics of the filler metal used for welding using both the classic MAG PULSE method and the high-performance MAG TANDEM method based on the manufacturer’s certificate are shown in Table 3.

The choice of the filler metal for welding was dictated by both its good plastic properties and ductility (Table 3) and the possibility of obtaining the desired hardness on the face side of the joint using the required heat input.

The welded joint was made under automated conditions as a V butt joint with a groove angle of 45° using the MAG PULSE and MAG TANDEM welding processes [22,23,24,25,26,27,28]. The MAG PULS welding process was used in the area of the first four reverse-side beads, while the rest of the weld groove was filled using the high-performance TANDEM version of the MAG process.

**Figure 1:**
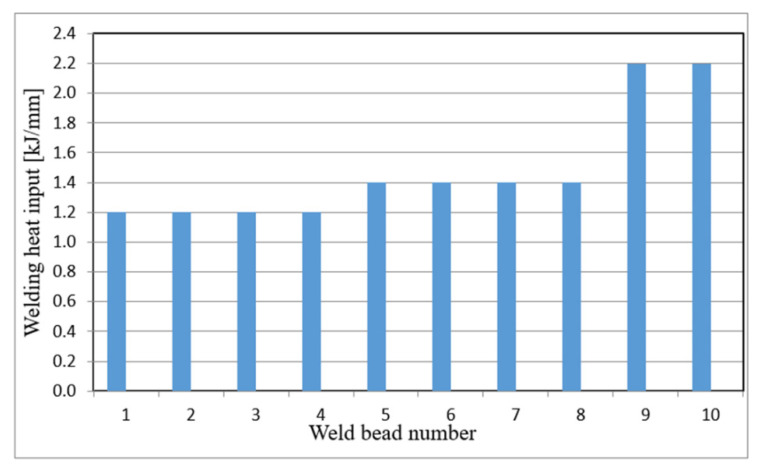
Distribution of heat input in individual beads of the welded joint.

The high-performance MAG TANDEM is a two-electrode process with an independent power supply for each electrode [22,23]; it allows the achievement of much higher welding efficiency compared to the conventional single-electrode process [26,27]. The welding tests were carried out using the Cloos equipment. For both methods, the SG3 welding electrode for advanced high-strength steels was used as a filler material in the welding process. The 1.2 mm electrode wire and the M21 shielding gas mixture were used [28]. The first four beads, including the reverse bead, and the next three ones were made by the classic MAG PULSE method using heat input of approx. 1.2 kJ/mm, while the other six fill passes were made by the high-performance MAG TANDEM method–the first four ones with a heat input of 1.4 kJ/mm and the last two ones, forming the weld face, with a heat input of 2.2 kJ/mm (Figure 1).

The heat input control in this area of the welded joint (Figure 1) was to provide a high-quality connection, while producing a joint that meets the required mechanical properties. The higher heat input in the weld face area (beads No. 9, 10) compared to the other areas of the weld (beads No. 1–8) allowed weld hardness similar to that of the materials being joined to be obtained. The use of the above heat inputs in the welding process has also resulted in obtaining a high-performance production process, which is economically advantageous.

In welding the first four beads, a straight technique was used, and for the other ones, the weaving technique with an oscillation width of 10 to 15 mm and a frequency of 0.6 Hz was applied. Before welding, the materials were pre-heated to a temperature between 140 and 160 °C in the welding zone.

At first, non-destructive tests, such as visual test (VT) according to EN ISO 17637:2017-02 [29], magnetic particle test (MT) according to EN ISO 17638:2017-01 [30] and ultrasonic test (UT) according to EN ISO 17640:2019-01 [31], were performed to evaluate the test welded joint. In MT, the Silver Yoke HD 230 AC/DC magnetic yoke flaw detector was used, while UT was performed using the Olympus EPOCH 650 flaw detector. The VT, MT and UT tests were performed over the entire length of the joint, on the weld face and the weld root side, in accordance with the requirements of the relevant standards [29,30,31,32]. For the evaluation of the test joint, quality level B, in accordance with the requirements of EN ISO 5817:2014-05 was adopted [33]. In the second stage of testing, according to EN ISO 15613-2006 [34], destructive tests, such as impact, macroscopic, hardness distribution and microscopic tests, were performed to evaluate the properties of the joint.

The impact test was performed using the Charpy V method on standard samples taken in three areas, i.e., weld and two HAZs–on the HTK 900H steel side and on the A6 casting side. In each of the three areas, the test samples were taken from the face and the root of the weld and from the central zone of the weld. The test was carried out at room temperature in accordance with EN ISO 148-1:2017-02 [35] using a WOLPERT Charpy V PW-15 pendulum. The impact strength was evaluated on three samples taken from each of the zones based on their average values according to the adopted criterion of KV_min_ 45 J as defined in EN ISO 15614-1:2017-08 [36].

The macroscopic examination was performed in accordance with EN ISO 17639:2013-12 [37] on the transverse microsection, which included the weld, both HAZs and adjacent base material areas of the welded joint. The sample was taken from the initial part of the weld in accordance with EN ISO 15614-1:2017-08 [36]. The surface of the microsection was etched with nital. The macrostructure of the welded joint was evaluated using a magnification of ×10 in accordance with the adopted quality level B criterion as defined in EN ISO 5817:2014-05 [33].

The hardness measurement was made using the Vickers HV10 method in accordance with EN ISO 9015-1:2011 [32]. The measurements were performed on the transverse microsection in three measurement lines determined next to the face of the weld, the root of the weld and in the central zone, respectively. Three measurements were taken along the measurement line in each of the welded joint zones (weld, HAZ and base materials). The measurements were evaluated according to the adopted criterion of HV10_max_ 450 as defined in EN ISO 15614-1:2017-08 [36]. The QATM Qness 60 A+ hardness tester was used for the test.

The microscopic examination was performed in accordance with EN ISO 17639:2013-12 [37] on the metallographic microsection, which included the weld, both HAZs and adjacent base material areas of the welded joint. The sample was taken from the initial part of the weld in accordance with EN ISO 15614-1:2017-08 [36]. The surface of the metallographic microsection was etched with nital. The microstructure of the welded joint was evaluated using optical microscopy with a Keyence VHX 7000 (OM) digital microscope and scanning electron microscopy (SEM) with a JEOL JSM-6610LV microscope. The chemical composition analysis of the base materials was carried out using a SpectroLab spark spectrometer.

## 3. Research Results and Analysis

### 3.1. Non-Destructive Testing

The visual testing revealed that the test joint was made correctly both on the weld penetration side and the weld face side. The evaluation of the joint meets the specified requirements in accordance with the adopted quality level B criterion. In turn, the magnetic particle testing revealed neither non-linear nor linear indications in both tested areas on the weld face and the weld root side. The evaluation of the joint also confirmed the quality level B. After obtaining positive results of the surface testing (VT and MT), the volumetric ultrasound testing of the test joint was performed. Based on the ultrasound testing, the test joint was qualified for quality level B.

The positive results obtained in non-destructive testing were the basis for further destructive testing to evaluate the properties of the test joint.

### 3.2. Destructive Testing

The impact test was performed using the Charpy V method on the standard samples at room temperature for the weld metal and two HAZs on the HTK 900H steel side and on the A6 casting side. For each zone, the samples were taken in three areas, i.e., on the weld face side, on the weld root side and in the central zone. The results of the impact tests are presented in Table 4.

In the impact test, all the results were positive and met the criterion of KV_min_ 45 J. The most favorable results were obtained for the heat-affected zone on the HTK 900H steel side where the average value of impact energy KV was 159 J. Lower values of impact energy were obtained for the heat-affected zone on the A6 casting side where the average KV was 86 J and a single result in this zone was 48 J; this lowest result was obtained for a sample taken from the HAZ next to the face of the weld. The average KV in the weld was 96 J. The impact energy results for the base materials themselves were adopted based on the manufacturers’ certificates and amounted to KV_−20_ 56 J for HTK 900H and KV_−20_ 78 J for A6, respectively.

The studies carried out so far have shown that welding Hardox steels results in an increase in their embrittlement within the welded joint area [7,13,38,39,40]. The impact energy of metallic alloys depends not only on their structure, but also on the grain size or the precipitation processes at the grain boundaries. For martensitic/bainitic structures, the crack resistance is related to the width of the laths/packets [41]. In [39], it has been demonstrated that the increase in the prior austenite grain size in Hardox 450 steel from 18 μm to 124 μm results in a more than a 3.5-time reduction in the impact energy. The significant prior austenite grain growth in Hardox steel which has a material impact on the properties of this group of steels is observed at above 1000 °C [42]. In turn, the Authors of [5] indicate that the time of cooling in the range of 800–500 °C to ensure the proper toughness of the welded joint should not be less than 3 s. The fracture toughness of high-strength steel joints is also significantly affected by the heat input; its value greater than 20–30 kJ/cm results in a significant reduction in the impact energy of the produced test joint not only in room but also at a reduced temperature [5]. Similar conclusions were presented by the Authors of [40]. The presence of the micro-addition of boron in the test steel (Table 2) can also contribute to the refinement and reduction in the size of martensitic packets and its refining effect on the grain/lath boundaries can improve the increase in the strength properties and fracture toughness [43]. According to [16], the presence of acicular ferrite in the microstructure of the joint also has an advantageous effect on the increase in the toughness of the steel. The high relative values of the impact energy obtained for the test joint (Table 4) are probably due to the presence of fine prior austenite grain in its microstructure and the related refinement of the size of martensite/bainite blocks and packets in HAZ and the presence of acicular ferrite in the microstructure, and for HTK 900H steel–also the micro-addition of boron. On the other hand, the diverse impact energy of the joint on its cross-section was related to the characteristic features of the materials being joined: HTK 900H–steel and A6–casting.

**Figure 2:**
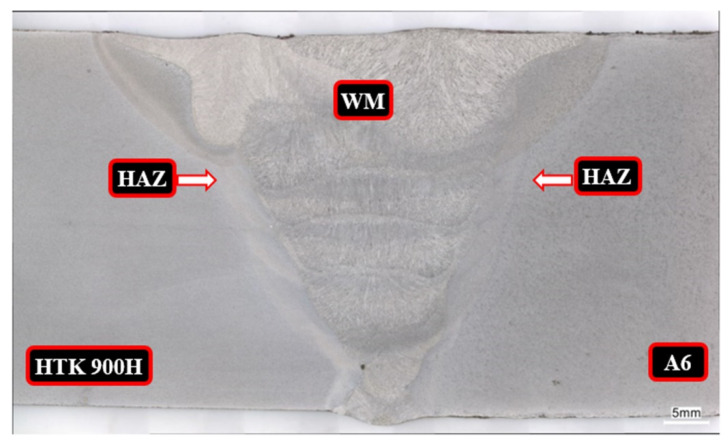
Macroscopic cross-section of HTK 900H/A6 casting welded joint, where: WM—weld metal; HAZ—heat affected zone; HTK 900H—HTK 900 steel; A6—A6 cast steel.

The macroscopic examination performed on the transverse microsection of the welded joint is presented in Figure 2. The macroscopic image of the test HTK 900H steel + A6 casting welded joint shows that the joint has the correct construction, i.e., correct weld penetration and regular fusion into both edges of the metals, proper arrangement of individual passes and beads, and small and mild weld face reinforcement. The HAZ width is equal on both sides of the fusion line and amounts to 3.5 mm in the bottom part of the weld and 6.0 mm in its top part at the height of the last layer. In addition, no significant welding imperfections were found on the cross-section of the joint in all the areas, i.e., base materials, heat affected zones (HAZs) and weld. The macroscopic examination of the welded joint between HTK 900H steel and A6 casting confirms that the connection was made correctly and with good quality.

The hardness measurement was made in three lines, i.e., near the face and the root of the weld and in the central zone, by making three indentations for each of the test zones (weld, HAZ and base materials). The results of the hardness measurement of the welded joint are presented in Table 5.

**Figure 3:**
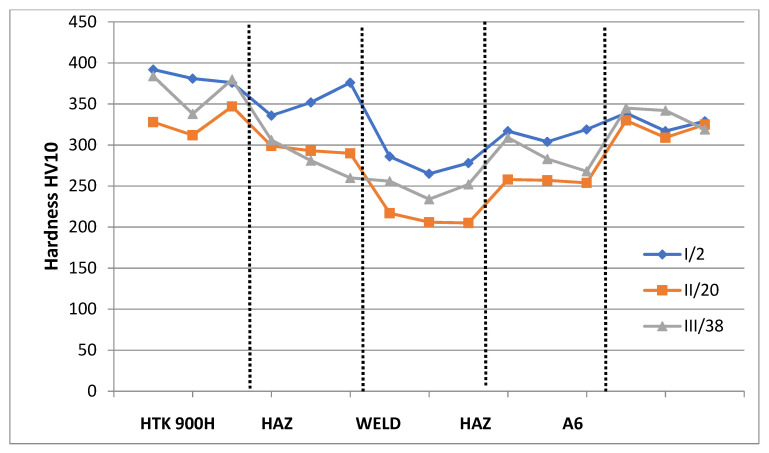
Hardness distribution on the cross-section of the welded joint.

The hardness of the test steel in the as-received condition was diverse and amounted to between 392 and 312 HV10, whereas the hardness of the A6 casting in the as-received condition varied between 345 and 309 HV10. The differences in the hardness of the base materials in the as-received condition resulted from both the difference in the chemical composition that favored the possibility of obtaining non-diffusive structures (martensite, bainite) and the technology for producing these details. The obtained results of hardness measurement of the welded joint meet the requirements for joints subjected to the welding procedure qualification in accordance with EN ISO 15614-1:2017-08 [36]. According to the requirements for this group of materials, the maximum hardness in the test joint should not exceed 450 HV [34]. The measurements revealed that the obtained hardness results did not exceed the adopted maximum value. The observed variations in the hardness of the test joint result from differences in the chemical composition of the base materials (their capacity for hardening), the effect of the temperature and cooling time in the specific HAZ and the filler metal used for welding. The highest hardness level of 336 to 392 HV10 was obtained for the first measurement line I/2 in the HTK 900H base material zone and the adjacent HAZ. The lowest hardness level of 265 and 286 HV10 for the first measurement line was obtained in the weld. In the latter base material, i.e., A6 casting, and in the adjacent HAZ, the hardness of 304 to 339 HV10 was obtained. For the other measurement lines (II/20 and III/38), the hardness results obtained are lower, particularly in the HTK 900H, weld and both HAZs. Only in the A6 casting zone, the hardness results are similar for all measurement lines. The hardness distribution in the test joint is well illustrated by the chart in Figure 3.

The reduction in hardness in HAZ compared to that of the base material on both the HTK 900H steel side and the A6 casting side was related to the phase changes in this area due to the introduction of thermal energy into the materials during the welding process. The increase in temperature in the HAZ area leads to the austenitizing (or partial austenitizing) process, following which, depending on the rate of cooling, the material-specific structures are obtained [11,44]. Further away from the fusion line, when the temperature is below A_c1_, the tempering process takes place, resulting in reducing the hardness. The lower hardness of the joined materials in HAZ was due to the grain size growth, the presence of bainite and diffusion structures (ferrite) and the occurrence of mixed structures. According to [45], the softening of martensite in low-alloy steels may also result from the recovery process of lath substructure and spheroidized cementite.

**Figure 4:**
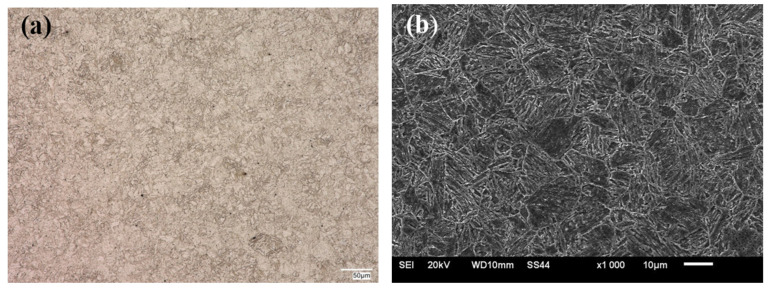
Microstructure of HTK 900H steel–base material of the test joint: (**a**) OM; (**b**) SEM.

**Figure 5:**
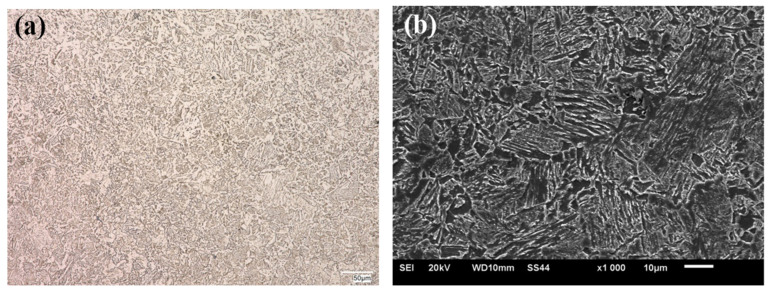
Microstructure of A6 casting, (**a**) OM; (**b**) SEM.

**Figure 6:**
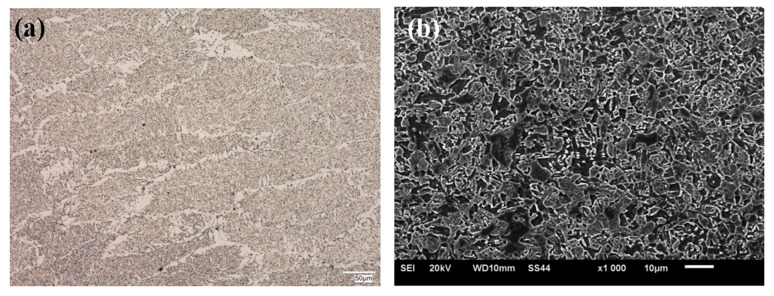
Microstructure of HAZ in HTK 900H steel, (**a**) OM; (**b**) SEM.

**Figure 7:**
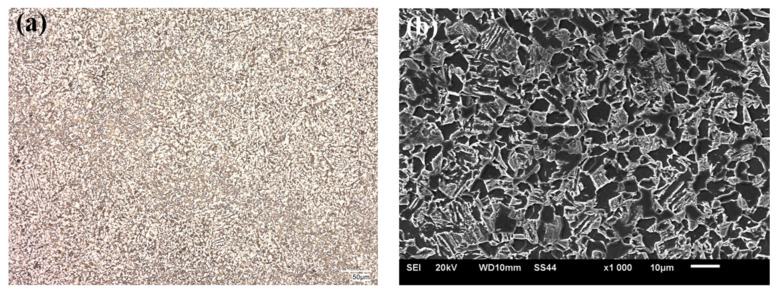
Microstructure of HAZ in A6 casting, (**a**) OM; (**b**) SEM.

**Figure 8:**
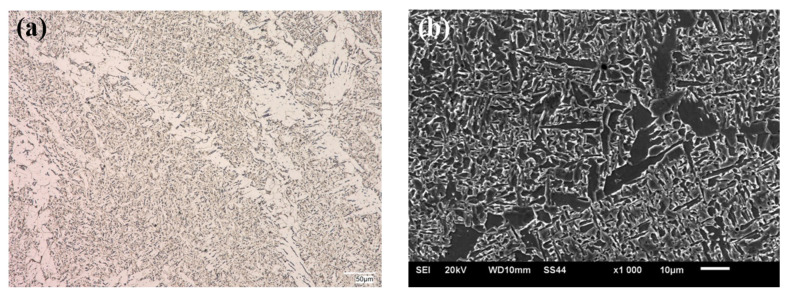
Microstructure of weld, (**a**) OM; (**b**) SEM.

The microstructural analysis was made for the zone next to the weld face of the HTK 900H steel + A6 casting welded joint, taking into account all areas of the joint, i.e., base materials, HAZs and weld. The example microstructural images of the test zones of the welded joint are shown in the following Figure 4, Figure 5, Figure 6, Figure 7 and Figure 8.

The HTK 900H base material had a fine-grained martensitic structure with dispersive precipitates, which were observed at the lath boundaries and at the prior austenite grain boundaries (Figure 4). The microstructure of the test steel is typical of this group of materials [3,10] and provides high strength properties and good abrasive wear resistance together with satisfactory plastic properties and impact load resistance (Table 1).

The A6 casting base material had a bainitic (or bainitic/martensitic) structure with single ferrite grains and precipitates. The precipitates were observed at the prior austenite grain boundaries as well as at the bainite/martensite boundaries and within the bainite/martensite laths (Figure 5). The resulting structures of the joined materials in the as-received condition were determined by their chemical composition and the production process.

The welding process, i.e., the introduction of energy into the material and the associated effect of temperature on the base material adjacent to the weld area, results in changes in the microstructure of the materials being joined and the formation of the heat-affected zone (HAZ). The HAZ has a diverse microstructure in its individual areas, which depends on the heating temperature of the given area, the cooling rate and the grade of the given alloy. Obtaining the specific microstructure in HAZ also depends on the welding process, the geometry of the weld joint, and also the morphology of inclusions or precipitates [5].

In the HAZ of HTK 900H steel, the martensitic/bainitic/ferritic structure was observed (Figure 6). Ferrite was precipitated at the grain boundaries in the allotriomorphic and partially lamellar form. The presence of ferrite in the microstructure of this area indicates that the analyzed area was heated up to the two-phase region (α + γ). The relatively small share of this phase in the structure suggests that the temperature of this area was close to A_c3_. The presence of acicular ferrite in the microstructure of the welded joint is extremely important from the point of view of its mechanical properties. Increasing the acicular ferrite ratio is an important phase as it increases the strength properties and toughness of the weld metal [16]. In [18], the microstructure examination was carried out on the Hardox 450/Strenx 700 steel joint and it was demonstrated that the structure in the HAZ of Hardox steel was changing on the cross-section of the joint from the coarse-grained martensitic/bainitic structure nearby the fusion line through the fine-grained martensitic/bainite and ferritic/bainitic structure to the structure corresponding to that of the base material.

In the HAZ of the welded joint on the A6 casting side, the structure was observed, which, similarly to the HTK steel, was dependent on the distance from the fusion line. Close to the fusion line, the bainitic structure with acicular ferrite was visible, while further away, the fine-grained ferritic/pearlitic microstructure was observed. In the microstructure of this area, degenerated pearlite was observed, which indicated accelerated cooling compared to the conventional rate of diffusive transformation. In addition, partial fragmentation of cementite plates was also observed in pearlite (Figure 7). The resulting microstructures of the welded base materials within HAZ were similar to those presented in the works on the issues relating to the welding of these construction materials, e.g. [5,11,14,16,18,40,46].

In the weld of the test joint, a dendritic bainitic structure with acicular and polygonal ferrite at the crystallization grain boundaries was observed (Figure 8). Obtaining this structure in the weld was determined by the filler material used, its chemical composition, welding parameters and cooling rate.

## 4. Conclusions

The research was conducted on a thick-walled joint of HTK 900H wear-resistant steel welded with an A6 cast profile using the SG3 filler metal. The joint was made in the lower root part using the MAG PULSE method, while the rest of the weld groove was made by the high-performance MAG TANDEM method under automated conditions. The performed tests of the dissimilar joint allowed the following conclusions to be drawn:

1. In the weld face region of the examined joint, the tests showed the occurrence of a structure with a dominant volume fraction of martensite/bainite ensuring the achievement of the expected properties.
2. The hardness of the examined joints in the weld face region in both of the Heat-Affected Zones (HAZ) was similar to that of the base materials and amounted to 383 HV10 for HTK 900H, from 336 to 376 HV10 for HAZ, whereas 328 HV10 for the A6 alloy, and from 304 to 319 HV10 for A6 alloy in HAZ. The lowest hardness value in the weld (276 HV10) results from applying the SG3 wire as additional material.
3. The obtained joint was characterized by high ductility. The energy of cracking in HAZ of the HTK 900H steel amounted to 159 J, in A6 alloy-86 J, and in the weld-159 J.
4. The use of the high-performance welding process carried out under automated conditions and the skilful heat input control produce a joint with both the required hardness, which ensures the abrasive wear resistance in the weld face area and the high resistance to dynamic loads.

## Figures and Tables

**Table 1:** Characteristics of HTK 900H and A6 base materials based on the certificates [19,20].

Material Type	Chemical Composition, % wt.									Mechanical Properties				
	C	Mn	Si	Cr	Mo	Ni	P	S	CEV	TS MPa	YS MPa	El. %	KV_−20_ J	HB
HTK 900H	0.15	1.25	0.31	0.73	0.21	0.03	0.025	0.01	0.45	1250	980	12	56	400
A6	0.28	1.30	0.24	-	-	-	0.028	0.01	0.46	870	720	13	78	300

Where: TS—tensile strength; YS—yield strength; El.—elongation; KV—impact energy; HB—Brinell hardness.

**Table 2:** Characteristics of HTK 900H and A6 base materials based on the chemical composition analysis.

Material Type	Chemical Composition, % wt.							
	C	Si	Mn	P	S	Cr	Mo	Nb
HTK 900H	0.17	0.31	1.28	0.012	0.005	0.74	0.23	0.03
A6	0.28	0.31	1.35	0.036	0.011	0.12	-	-

**Table 3:** Characteristics of SG3 filler metal based on the manufacturer’s certificate [21].

Material Type	Chemical Composition, % wt.						Mechanical Properties			
	C	Mn	Si	Cr	Mo	Ni	TS MPa	YS MPa	El. %	KV_−20_ J
SG3	0.08	1.64	0.88	0.03	0.01	0.04	620	500	26	80

Where: TS—tensile strength; YS—yield strength; El.—elongation; KV—impact energy.

**Table 4:** Results of impact tests for HTK900H + A6 casting welded joint.

No.	Sample Designation	Sample Dimensions			Impact Energy		Fracture Appearance	Rating
		a [mm]	b [mm]	S [mm^2^]	KV [J]	Average KV		
1	BW40/VWT 0/28-1	10	8	80	95	96	nd	**A**
2	BW40/VWT 0/14-2	10	8	80	76		nd	
3	BW40/VWT 0/2-3	10	8	80	118		nd	
4	BW40/VHT H 0/28-1	10	8	80	158	159	nd	**A**
5	BW40/VHT H 0/14-2	10	8	80	184		nd	
6	BW40/VHT H 0/2-3	10	8	80	136		nd	
7	BW40/VHT A 0/28-1	10	8	80	85	86	nd	**A**
8	BW40/VHT A 0/14-2	10	8	80	124		nd	
9	BW40/VHT A 0/2-3	10	8	80	48		nd	

Abbreviations: nd—no defects; **A**—acceptable; NA—not acceptable, H—HTK900H; A—A6.

**Table 5:** Results of hardness distribution for HTK 900H + A6 casting welded joint.

No.	Sample Designation	Measurement Line No.	Results					*Acceptance*	
			BM1	HAZ1	S	HAZ2	BM2	YES	NO
1	HV10/BW40 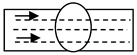	I/2	392	336	286	317	339	**X**	**-**
			381	352	265	304	317		
			376	376	278	319	329		
		II/20	328	299	217	258	330		
			312	293	206	257	309		
			347	290	205	254	325		
		III/38	384	306	256	309	345		
			338	281	234	283	342		
			380	260	252	268	319		
BM1-HTK 900H					BM2-A6				

Where: **I/2**—the first hardness measurement line at a distance of up to 2 mm from the top surface of the joint (on the weld face side); **II/20**—the second hardness measurement line at a distance of 20 mm from the top surface of the joint (middle zone); **III/38**—the third hardness measurement line at a distance of 38 mm from the top surface of the joint (root zone).

## Data Availability

Not applicable.
